# LncRNA CCAT2 promoted osteosarcoma cell proliferation and invasion

**DOI:** 10.1111/jcmm.13518

**Published:** 2018-03-04

**Authors:** Lihua Yan, Xiangkun Wu, Xianzhe Yin, Feng Du, Yongxi Liu, Xunmeng Ding

**Affiliations:** ^1^ Department of Oncology The Nanyang Second People's Hospital Nanyang China; ^2^ Department of Orthopaedic The Nanyang Second People's Hospital Nanyang China

**Keywords:** osteosarcoma, long non‐coding RNAs, CCAT2

## Abstract

Long non‐coding RNA (lncRNA) plays important roles in tumour progression. Accumulating studies demonstrated that lncRNA colon cancer‐associated transcript 2 (CCAT2) acted as an oncogene in many tumours. However, the role of CCAT2 in the development of osteosarcoma has not been elucidated. In our study, we indicated that CCAT2 expression was up‐regulated in osteosarcoma tissues and cell lines (SOSP‐9607, MG‐63, U2OS and SAOS‐2). In addition, osteosarcoma cases with higher CCAT2 expression had a poorer disease‐free survival and shorter the overall survival time compared to those with lower expression. Overexpression of CCAT2 promoted osteosarcoma cell proliferation, invasion and cell cycle. Furthermore, ectopic expression of CCAT2 increased the expression of mesenchymal markers N‐cadherin, vimentin and snail and reduced the expression of N‐cadherin marker E‐cadherin. CCAT2 overexpression promoted the LATS2 and c‐Myc expression in osteosarcoma cell. These data indicated that CCAT2 served as an oncogene in osteosarcoma and promoted osteosarcoma cell proliferation, cell cycle and invasion.

## Introduction

Osteosarcoma is the most common bone tumour that mainly affects children and young adults 1, 2, 3, 4. Although many therapeutic strategies such as adjuvant chemotherapy, radiotherapy and wide tumour excision have been developed, the prognosis of osteosarcoma patients remains unsatisfied 5, 6, 7, 8, 9. Approximately half of the osteosarcoma patients eventually develop metastases, with pulmonary metastasis the most common cause 10, 11, 12, 13. Hence, it is essential to identify new biomarkers and novel treatment strategies for osteosarcoma.

lncRNAs are a member of the ncRNA family with longer than 200 nt in length 14, 15, 16, 17. Increasing studies has shown that lncRNAs play important roles in many cell biological processes including cell development, proliferation, apoptosis, migration and invasion 18, 19, 20, 21. LncRNAs are aberrantly expressed in various tumours such as breast cancer, lung cancer, hepatocellular carcinoma, bladder cancer, nasopharyngeal carcinoma 22, 23, 24, 25, 26. Growing studies also showed that lncRNAs play crucial roles in the development of osteosarcoma. For example, Wang *et al*. 27 showed that MALAT1 was overexpressed in osteosarcoma and ectopic expression of MALAT1 promoted proliferation and metastasis through inhibiting miR‐144‐3p expression in the osteosarcoma cells. Cai *et al*. 28 indicated that the expression of HNF1A‐AS1 was overexpressed in the osteosarcoma samples and knockdown of HNF1A‐AS1 suppressed cell cycle, migration and invasion in the osteosarcoma cell. Recently, lncRNA CCAT2 has been demonstrated to be up‐regulated in hepatocellular carcinoma, lung cancer and gastric cancer 29, 30, 31. Ectopic expression of CCAT2 promoted cell proliferation, migration and proliferation in malignancies 32, 33, 34, 35. In addition, Wu *et al*. showed that CCAT2 expression was elevated in breast cancer tissues and down‐regulation of CCAT2 suppressed the breast cancer invasion, proliferation and migration by inhibiting TGF‐β, Smad2 and α‐SMA expression 36. However, the role of CCAT2 in the development of osteosarcoma remains unknown.

In this study, we aimed to investigate CCAT2 expression level in osteosarcoma tissues and cell lines. We also examined the role of CCAT2 in osteosarcoma cell migration, proliferation and cycle. We demonstrated that CCAT2 expression was elevated in osteosarcoma tissues and cell lines. Overexpression of CCAT2 promoted the osteosarcoma cell proliferation and invasion, indicating that CCAT2 acted an oncogenic role in osteosarcoma.

## Materials and methods

### Tissue samples

The forty osteosarcoma tissues and pair adjacent normal tissues were collected from our department. None of these patients received chemoembolization, radiotherapy and percutaneous ablation before surgery. The Ethics Committee of Nanyang Second People's Hospital has approved this study, and written informed consent from all patients was obtained. The characteristics of these patients were described in Table S1.

### Cell culture and transfection of cell lines

The human osteosarcoma cell lines SOSP‐9607, MG‐63, U2OS and SAOS‐2 and one osteoblast cell line (hFOB) were purchased from American Type Culture Collection. These cells were cultured in the DMEM (Dulbecco's modified Eagle's medium) with foetal bovine serum (FBS, Gibco, Grand Island, NY, USA), streptomycin and penicillin. pcDNA‐CCAT2 and control were synthesized from GenePharma (Shanghai, China). These cells were transfected with pcDNA‐CCAT2 or control vector using the Lipofectamine 2000 (Invitrogen, Carlsbad, CA, USA) following to protocol.

### RNA extraction and qPCR analysis

Total RNA from cultured cells or tissues was extracted using the TRIzol (Invitrogen) following to manufacturers’ protocol. qRT‐PCR (quantitative real‐time PCR) was used to determine the lncRNA and mRNA expression using SYBR (Takara, China) on the 7900HT system. The level expression of mRNA and lncRNA was normalized to the GAPDH and U6, respectively, and the fold change was measured by the value (2^−ΔΔCT^) method. The special primers for CCAT2, forward: 5′‐AGACAGTGCCAGCCAACC‐3′, reverse: 5′‐TGCCAAACCCTTCCCTTA‐3′; LATS2, forward: 5′‐ACCCCAAAGTTCGGACCTTAT‐3′, reverse: 5′‐CATTTGCCGGTTCACTTCTGC‐3′; E‐cadherin, forward: 5′‐TCT TCCAGGAACCTCTGTGATG‐3′, reverse: 5′‐CAATGCCGCCATCGCTTACACC‐3′; c‐Myc, forward: 5′‐CGGTTTGTCAAACAGTACTGCTACGGAG‐3′, reverse: 5′‐CTCAGCCGTCCAGACCCTCGCATTATAAAG‐3′; N‐cadherin, forward: 5′‐CCGGAGAACAGTCTCCAACTC‐3′, reverse: 5′‐CCCACAAAGAGCAGCAGTC‐3′; and GAPDH, forward: 5′‐AATGGACAACTGGTCGTGGAC‐3′, reverse: 5′‐CCCTCCAGGGGATCTGTTTG‐3′.

### Cell proliferation, cell cycle and invasion

The MG‐63 cells were cultured in the 96‐well plate and cultured for 24, 48 and 72 hrs after transfection. Cell Counting Kit CCK 8 analysis was performed to determine the cell proliferation. The absorbance at 450 nm was detected on the microplate reader (USA). For cell cycle assay, cell was fixed in ethanol (70%) for 1 hr and then incubated with RNase A. Subsequently, cell was stained with propidium iodide (Becton‐Dickinson, San Jose, CA, USA) and determined using flow cytometer BD FACScan (San Jose, CA, USA). For cell invasion analysis, the BD Matrigel chamber (BD Biosciences, UK) was used. The cell was seeded onto the membrane chamber in the serum‐free medium, and medium with 10% FBS was added to the bottom chamber. After 24 hrs, the cell in the low chamber was stained with crystal violet (Sigma‐Aldrich, St. Louis, MO, USA) and counted.

### Western blot

Total protein from established cells and tissues was prepared and determined using the BCA (Thermo, PA, USA). Total protein was resolved on the 12% SDS–PAGE and transferred to a PVDF membrane. The membrane was blocked with dry milk and immunostained with the primary antibodies (E‐cadherin, N‐cadherin, vimentin and snail, dilution 1:2000 and GAPDH, dilution 1:5000, Abcam, Cambridge, UK) at 4°C overnight. After incubated with the secondary antibody, the signal was visualized by chemiluminescent detection system (Pierce, Thermo, PA, USA).

### Statistical analysis

All statistical assays were measured by spss 17.0 software (IBM, Chicago, IL, USA). Data were formulated as mean ± S.D. The difference between groups was analysed by the Student's *t*‐test. Statistical significance was considered as *P* < 0.05.

## Results

### CCAT2 expression was elevated in osteosarcoma tissues and cell lines

**Figure 1:**
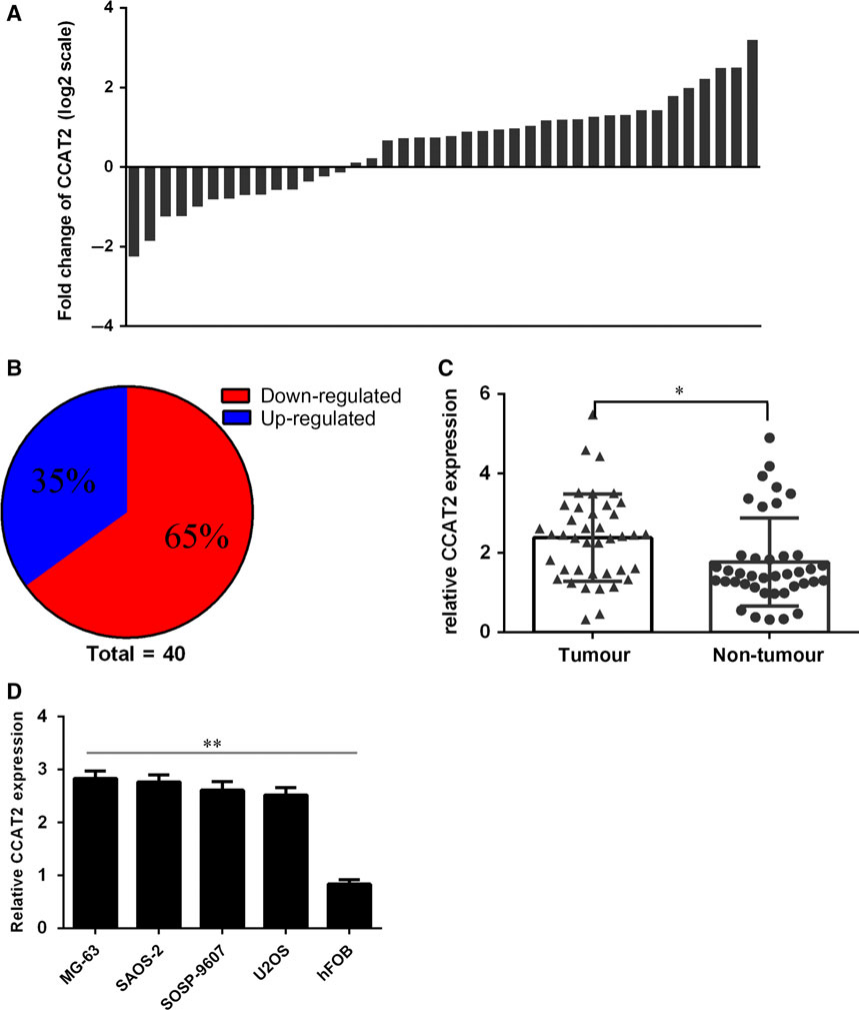
Colon cancer‐associated transcript 2 (CCAT2) was up‐regulated in the osteosarcoma tissues and cell lines. (**A**) Relative expression level of CCAT2 expression in osteosarcoma tissues and adjacent non‐tumour tissues was analysed by qRT‐PCR. Data were shown as log 2 of fold change in osteosarcoma tissues relative to non‐tumour adjacent tissues. (**B**) The expression of CCAT2 was up‐regulated in 26 (26/40, 65%) osteosarcoma cases. (**C**) CCAT2 expression level was higher in the osteosarcoma tissues than in the control normal tissues. (**D**) The expression of CCAT2 was up‐regulated in the osteosarcoma cell lines (SOSP‐9607, MG‐63, U2OS and SAOS‐2) compared to the one normal osteoblast cell line (hFOB). GAPDH was used the internal control. *P < 0.05 and **P < 0.01.

We firstly detected CCAT2 expression in osteosarcoma tissues. As shown in Figure 1A and B, the expression of CCAT2 was up‐regulated in 26 osteosarcoma cases (26/40, 65%) compared with the normal adjacent tissues. In general, CCAT2 expression level was higher in osteosarcoma tissues than in the normal control tissues (Fig. 1C). CCAT2 expression was up‐regulated in osteosarcoma cell lines (SOSP‐9607, MG‐63, U2OS and SAOS‐2) compared to the normal osteoblast cell line (hFOB) (Fig. 1D).

### High CCAT2 expression was correlated with poor survival

**Figure 2:**
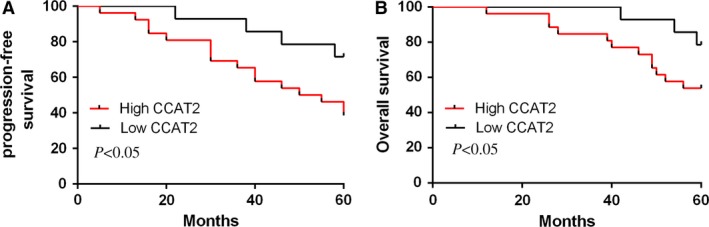
High expression of CCAT2 was correlated with poor survival. (**A**) Osteosarcoma cases with the higher CCAT2 expression had a poor disease‐free survival time compared to those with the lower expression. (**B**) Osteosarcoma cases with the higher CCAT2 expression had a poor overall survival time compared to those with the lower expression.

Osteosarcoma cases with the higher CCAT2 expression had a poorer disease‐free survival and shorter overall survival time compared to those with the lower expression. CCAT2 was an independent prognostic factor for disease‐free survival (Fig. 2A) and the overall survival time (Fig. 2B) for osteosarcoma cases.

### Overexpression of CCAT2 promoted cell proliferation and cycle in osteosarcoma

**Figure 3:**
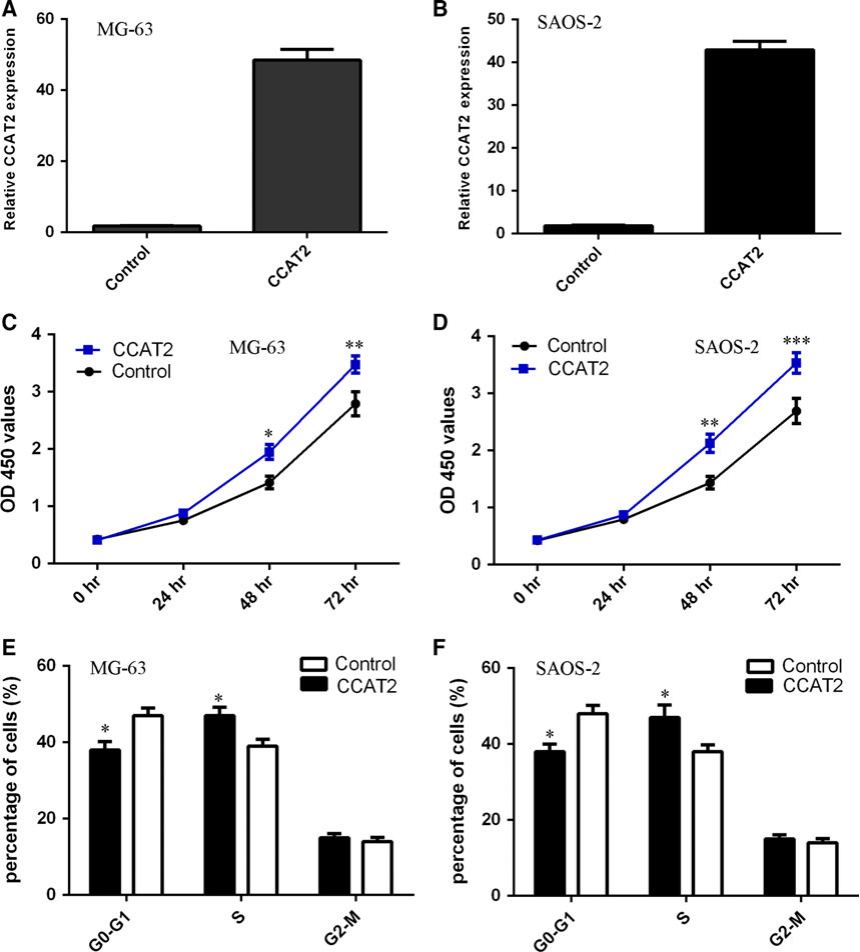
Overexpression of CCAT2 promoted osteosarcoma cell proliferation and cycle. (**A**) The expression of CCAT2 was measured by qRT‐PCR in the MG‐63 cell. The expression of CCAT2 was significantly up‐regulated in the MG‐63 cell treated with pcDNA‐CCAT2 compared to the cells treated with empty vector. (**B**) The expression of CCAT2 was measured by qRT‐PCR in the SAOS‐2 cell. The SAOS‐2 cells were transfected with pcDNA‐CCAT2 or empty vector, respectively. (**C**) Ectopic expression of CCAT2 enhanced the MG‐63 cell proliferation. The MG‐63 cells were transfected with pcDNA‐CCAT2 or empty vector, respectively. (**D**) Overexpression of CCAT2 promoted the SAOS‐2 cell proliferation. The SAOS‐2 cells were transfected with empty vector were used as the control. (**E**) Elevated expression of CCAT2 increased the percentages of cells in the S phase in the MG‐63 cell. The MG‐63 cells were transfected with pcDNA‐CCAT2 or empty vector, respectively. (**F**) Elevated expression of CCAT2 increased the percentages of cells in the S phase in the SAOS‐2 cells. The SAOS‐2 cells were transfected with empty vector were used as the control. *P < 0.05,**P < 0.01,***P < 0.001.

The expressions of CCAT2 were increased in both MG‐63 and SAOS‐2 cells after transfected with the pcDNA‐CCAT2 (Fig. 3A and B). Ectopic expression of CCAT2 enhanced cell proliferation in MG‐63 and SAOS‐2 proliferation (Fig. 3C and D). Elevated expression of CCAT2 increased the percentages of cells in the S phase in both MG‐63 (Fig. 3E) and SAOS‐2 cell lines (Fig. 3F).

### Ectopic expression of CCAT2 promoted osteosarcoma cell invasion

**Figure 4:**
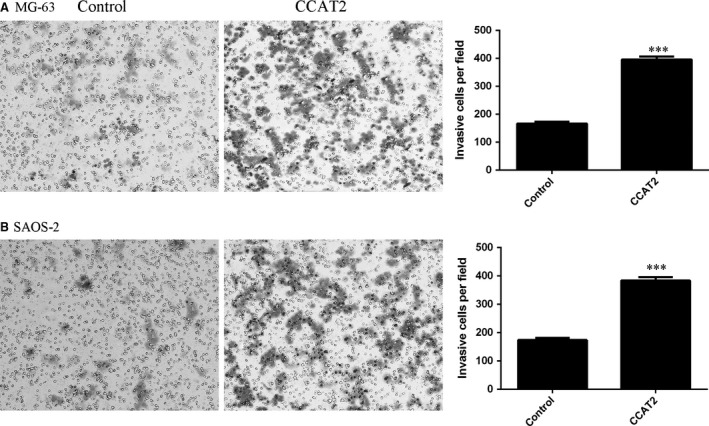
Ectopic expression of CCAT2 promoted the osteosarcoma cell invasion. (**A**) Elevated expression of CCAT2 enhanced the MG‐63 cell invasion. The relative invasive cells were shown. The MG‐63 cells were transfected with empty vector were used as the control. (**B**) Overexpression of CCAT2 promoted the SAOS‐2 cell invasion. The relative invasive cells were shown. The SAOS‐2 cells were transfected with empty vector were used as the control. ***P < 0.001.

To study whether CCAT2 was involved in the osteosarcoma metastasis, we measured the effects of CCAT2 on the invasion of the osteosarcoma cells. Ectopic expression of CCAT2 enhanced the MG‐63 cell invasion using Matrigel invasion assays (Fig. 4A). Overexpression of CCAT2 promoted SAOS‐2 cell invasion (Fig. 4B).

### CCAT2 overexpression increased epithelial–mesenchymal transition (EMT) progression

**Figure 5:**
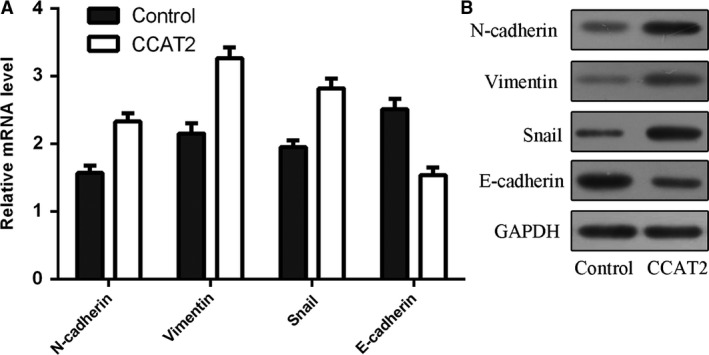
Colon cancer‐associated transcript 2 (CCAT2) overexpression increased epithelial–mesenchymal transition (EMT) progression. (**A**) The mRNA expression of N‐cadherin, vimentin, snail and E‐cadherin was measured by qRT‐PCR in the MG‐63 cell. CCAT2 overexpression promoted the mRNA expression of N‐cadherin, vimentin and snail and decreased the mRNA expression of epithelial maker E‐cadherin. (**B**) The protein expression of N‐cadherin, vimentin, snail and E‐cadherin was measured by Western blot in the MG‐63 cell. Elevated expression of CCAT2 increased the protein expression of N‐cadherin, vimentin and snail and decreased the protein expression of epithelial maker E‐cadherin.

CCAT2 overexpression promoted the expression levels of N‐cadherin, vimentin and snail, which were the mesenchymal markers. Moreover, elevated expression of CCAT2 decreased the expression of epithelial maker E‐cadherin (Fig. 5A and B).

### CCAT2 overexpression promoted LATS2 and c‐Myc expression

**Figure 6:**
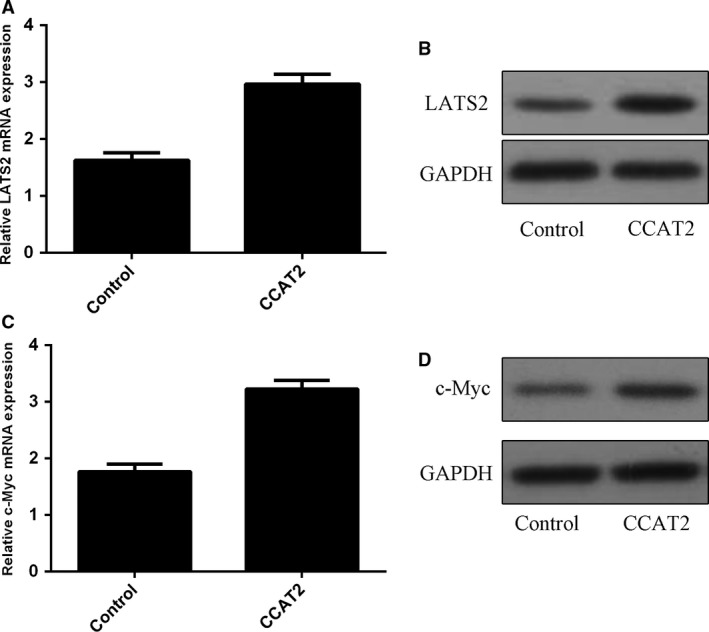
Colon cancer‐associated transcript 2 (CCAT2) overexpression promoted the LATS2 and c‐Myc expression. (**A**) CCAT2 overexpression increased the LATS2 mRNA expression in the MG‐63 cell using qRT‐PCR. The MG‐63 cells were transfected with empty vector were used as the control. (**B**) The protein expression of LATS2 was measured by Western blot in the MG‐63 cell. Elevated expression of CCAT2 promoted the protein expression of LATS2. The MG‐63 cells were transfected with empty vector were used as the control. (**C**) CCAT2 overexpression increased the c‐Myc mRNA expression in the MG‐63 cell. The MG‐63 cells were transfected with empty vector were used as the control. (**D**) The protein expression of c‐Myc in the MG‐63 cell was measured by Western blot. Overexpression of CCAT2 promoted the c‐Myc protein expression. The MG‐63 cells were transfected with empty vector were used as the control.

Overexpression of CCAT2 increased LATS2 expression in MG‐63 cell (Fig. 6A and B). Moreover, overexpression of CCAT2 promoted the expression of c‐Myc in MG‐63 cell (Fig. 6C and D).

## Discussion

LncRNAs act significant roles in the initiation and progression of tumour, and dysregulation of some lncRNAs may contribute to the tumorigenesis 37, 38, 39, 40. In this study, we indicated that CCAT2 expression was up‐regulated in osteosarcoma tissues and cell lines (U2OS, MG‐63, SOSP‐9607 and SAOS‐2). Higher CCAT2 expression indicated a poorer disease‐free survival and shorter overall survival time. Overexpression of CCAT2 promoted osteosarcoma cell proliferation, invasion and cell cycle. Furthermore, ectopic expression of CCAT2 increased the expression levels of mesenchymal markers vimentin, N‐cadherin and snail and reduced the expression of E‐cadherin. Moreover, CCAT2 overexpression promoted the expression of LATS2 and c‐Myc. These data suggested that CCAT2 served as an oncogene in osteosarcoma and promoted cell osteosarcoma proliferation, cell cycle and invasion.

Recent studies have indicated that CCAT2 plays important roles in several tumours. For instance, Redis *et al*. 41 showed that CCAT2 expression was overexpressed in breast cancer tissues and CCAT2 overexpression promoted breast cancer cell migration and decreased chemosensitivity to 5‐FU. Qiu *et al*. 42 demonstrated that CCAT2 expression was elevated in non‐small cell lung cancer tissues and higher expression of CCAT2 was correlated with poorer prognosis in lung adenocarcinoma patients. Reduced expression of CCAT2 inhibited lung cancer cell proliferation and invasion. Wang *et al*. 43 indicated that CCAT2 expression was up‐regulated in gastric cancer tissues. Patients with higher CCAT2 expression had shorter progression‐free survival and overall survival. Zeng *et al*. 33 proved that CCAT2 expression was up‐regulated in glioma cell lines and tissues. Patients with higher expression of CCAT2 showed poorer prognosis and survival. Knockdown CCAT2 expression decreased glioma cell growth, migration and invasion. However, the functional role and expression pattern of CCAT2 in osteosarcoma have not been elucidated. In this study, we firstly analysed the expression of CCAT2 in osteosarcoma tissues. We found that CCAT2 expression was up‐regulated in 26 osteosarcoma cases (26/40, 65%) compared with the adjacent tissues. Furthermore, the expression of CCAT2 was up‐regulated in osteosarcoma cell lines (MG‐63, SOSP‐9607, U2OS and SAOS‐2) compared to the hFOB. In addition, osteosarcoma cases with higher CCAT2 expression had a poorer disease‐free survival and shorter overall survival time compared to those with lower CCAT2 expression.

Next, we investigated the functional role of CCAT2 in osteosarcoma cell. We showed that CCAT2 overexpression increased cell proliferation in both MG‐63 and SAOS‐2 cells. Elevated expression of CCAT2 increased cell percentages in the S phase while decreased cell percentages in the G0‐G1 phase. Ectopic expression of CCAT2 promoted osteosarcoma cell invasion and increased the expression of mesenchymal markers N‐cadherin, vimentin and snail mRNA and decreased the expression of N‐cadherin marker E‐cadherin. LATS2 and c‐Myc are two important regulators that promote the cancer progression and involve in tumorigenesis 44, 45. Previous studies showed that CCAT2 overexpression promoted LATS2 expression in gastric cancer cell 46. In addition, Guo *et al*. 35 demonstrated that CCAT2 increased c‐Myc expression in glioma cells. Xu *et al*. 47 demonstrated that miR‐33b inhibited osteosarcoma cell invasion, proliferation and migration through suppressing the c‐Myc expression. In line with these, we also found that overexpression of CCAT2 promoted the LATS2 and c‐Myc expression.

In summary, the data from our study showed that CCAT2 expression was up‐regulated in osteosarcoma tissues and cell lines. Patients with higher CCAT2 expression demonstrated a poorer disease‐free survival and shorter overall survival time. Ectopic expression of CCAT2 promoted osteosarcoma cell proliferation, cell cycle and invasion and EMT progression. These results indicated that CCAT2 played an oncogenic role in the development and progression of osteosarcoma.

## Supporting information


**Table S1**. Clinicopathologic charateristics of patients with osteosarcoma.
