# Supplementary material for: LncRNA CCAT2 promoted osteosarcoma cell proliferation and invasion
Source: J Cell Mol Med. 2018 Mar 4;22(5):2592–9. doi: 10.1111/jcmm.13518 (PMC5908115; doi:10.1111/jcmm.13518)
Supplement: Supplementary file 1 — Table S1. Clinicopathologic charateristics of patients with osteosarcoma. [file JCMM-22-2592-s001.doc]

Table S1 Clinicopathologic charateristics of patients with osteosarcoma

| Parameter | Total  samples | Percentage | CCAT2 expression  Low High | | P |
| --- | --- | --- | --- | --- | --- |
| Age (years) |  |  |  |  | p >0.05 |
| ≥13 | 17 | 42.5% | 11 | 6 |  |
| <13 | 23 | 57.5% | 15 | 8 |  |
| Gender |  |  |  |  | p >0.05 |
| Male | 21 | 52.5% | 14 | 7 |  |
| Female | 19 | 47.5% | 12 | 7 |  |
| Location |  |  |  |  | p >0.05 |
| Femur | 20 | 50% | 12 | 8 |  |
| Tibia | 15 | 37.5% | 10 | 5 |  |
| Humeral bone | 4 | 10% | 3 | 1 |  |
| other | 1 | 2.5% | 1 | 0 |  |
| Pathological facture |  |  |  |  | p >0.05 |
| Present | 6 | 15% | 5 | 1 |  |
| Absent | 34 | 85% | 21 | 13 |  |
| Metastasis |  |  |  |  | p<0.01 |
| Present | 9 | 22.5% | 2 | 7 |  |
| Absent | 31 | 77.5% | 24 | 7 |  |
| Subtype |  |  |  |  | p >0.05 |
| Conventional | 18 | 45% | 12 | 6 |  |
| Non-conventional | 22 | 55% | 14 | 8 |  |
